# Knowledge assessment of women of reproductive age on birth defects: a descriptive cross-sectional study in Kenya

**DOI:** 10.11604/pamj.2024.48.79.43037

**Published:** 2024-06-28

**Authors:** George Nyadimo Agot, Joseph Kibuchi Wang´ombe, Marshal Mutinda Mweu

**Affiliations:** 1 Department of Public and Global Health, Faculty of Health Sciences, University of Nairobi, Nairobi, Kenya

**Keywords:** Knowledge, assessment, birth defects, women of reproductive age, cross-sectional study, Kenya

## Abstract

**Introduction:**

birth defects are defined as structural or functional congenital malformations occurring during intrauterine and detectable prenatally, at birth, or later. Birth defects-awareness creation among women of reproductive age would help in preventing the occurrence of birth defects of known aetiology worldwide. Thus, this study aimed to assess the birth defects knowledge of women of reproductive age.

**Methods:**

we adopted a descriptive cross-sectional study design in eleven purposively selected public hospitals. The study population comprised women with children under five years, and attending child-welfare clinics at the study hospitals. Descriptive analyses consisting of means, standard deviations, medians, and ranges were used to summarize continuous variables, whereas, percentages and proportions were used to summarize categorical variables.

**Results:**

the median age of the study participants was 26 years with a mean of 27 (Standard Deviation=5, Range=17-42). A majority (77%) achieved at least a secondary level of education, while the median gravidity was 2 with a mean of 2 (Standard Deviation=1, Range; 1-8). The study participants' knowledge was above average (67%), implying in every 10 of reproductive age 3 had sub-optimal knowledge of birth defects.

**Conclusion:**

women of reproductive age were substantially deficient in birth defects knowledge in the county. Thus, we would like to recommend to public health policymakers and health care providers to formulate short health messages on birth defects tailored to women attending child welfare and antenatal clinics at all levels of health care including community health services in the county.

## Introduction

Birth defects are defined as structural or functional congenital malformations occurring during intrauterine and detectable prenatally, at birth, or later in life attributable to identifiable environmental teratogens, identifiable genetic factors, as well as complex genetic and idiopathic environmental teratogens referred to as multifactorial inheritance [1,2]. The causes of more than two-thirds of birth defects are unknown, whereas the causes of the remaining one-third are known and attributed to known genetic and modifiable factors [1,3-5]. Birth defects-awareness creation and birth defects awareness-raising among women of reproductive age would certainly help in preventing the occurrence of birth defects of known aetiology worldwide. Birth defects are associated with childhood mortality, and morbidity as well as lifelong physical disabilities [1,2]. Birth defects contribute to greater proportions of childhood mortality despite the downward trend attributed to immunizations, control of diarrheal diseases, as well as acute respiratory tract infections, and improvements in healthcare services worldwide [6]. They have been noted as responsible for approximately 30% to 50% of perinatal, neonatal, and childhood mortality in developed settings, and 5% to 7% in developing countries, and the trend is still on an upward trajectory [6]. Resource-constrained settings may not have advanced biochemical and imaging birth defects detection techniques underscoring the disparity of mortality and morbidity associated with birth defects between developed and developing countries [7-10]. The prevalence of birth defects is however equally proportionate in both developed and developing countries [1,3]. Undoubtedly, this observation calls for sustained innovative low-cost effective public health strategies aimed at preventing and controlling the occurrence of birth defects to reverse this spiraling trend of birth defects globally.

Studies carried out in Kenya revealed an upward trend of major external structural birth defects in Kiambu County [11], associated with familial history of birth defects, or multifactorial inheritance and maternal residence at conception pointing to known genetic etiology, and maternal exposure to environmental teratogens, respectively [12]. Birth defect knowledge of women of reproductive age before conception would help in preventing the occurrence of these defects. Engaging primary preventive strategies during preconception aimed at preventing teratogen-induced birth defects such as congenital syphilis and rubella, environment-induced teratogens such as orofacial clefts attributed to maternal exposure to cigarette smoking, iodine deficiency-induced birth defects, folates deficiency-induced birth defects such as neural tube defects, advanced maternal age-related chromosomal disorders such as Down Syndrome, genetic counselling and screening of families at risk of birth defects are of great public health importance in reversing the upward trend of birth defects globally [1,3,13-25]. Public health preventive strategies for birth defects targeting pregnant women are similarly useful, though only if such women begin antenatal care utmost four weeks following cessation of regular menstruations. This would purposely allow these women to begin folic intake, multivitamin supplementation, avoid teratogenic medicines, teratogenic-environmental exposures, early treatment of teratogenic infections such as syphilis, and immunization against rubella among other strategies. Nonetheless, the aforementioned strategies would otherwise not be of optimal benefit in preventing and controlling birth defects because approximately half of pregnancies are usually not planned or unintended, and mostly recognized at the end of the first trimester (16) weeks when organogenesis is complete largely at eight weeks of gestation [1,3,7,26,2,7]. Thus, assessing birth defect knowledge of women of reproductive age underscores this study and underpins birth defect awareness-creation and awareness-raising among women of reproductive age. Additionally, this study could influence public health policy formulation and preventive strategies tailored to antenatal, maternal, child welfare care, and other health care services including community health services.

## Methods

### Study design and settings

This was a descriptive cross-sectional study conducted to assess the knowledge of women of reproductive age on birth defects. The study population consisted of women with children aged under five years, and attending child-welfare clinics at all (eleven) public hospitals in Kiambu County in Kenya purposively selected. The hospitals comprised three level five hospitals (Gatundu, Thika, and Kiambu) and eight-level four hospitals (Karuri, Kigumo, Kihara, Lari, Lussigetti, Nyathuna, Tigoni, and Wangige). The choice of cross-sectional study design for this study was premised on its ability to provide a snapshot of the knowledge of women of reproductive age on birth defects, whilst, the study hospitals were purposively chosen for being high volume hospitals by their nature thus increasing the number of the accessible study population. This was an observational study thus reported as per STROBE’s guidelines [28].

### Sample size determination

we used the Charan J and Biswas T formula for estimating sample size for a cross-sectional study stated below [29].


$$N=\frac{{Z_{1-\alpha /2}}^{2}p\left(1-p\right)}{d^{2}}$$

N = is the sample size estimate;

Z_(1-α/2)_ is standard normal variate at 5% type 1 error (p<0.05) usually set at 1.96 and 1% type 1 error (p<0.01) usually set at 2.58; we adopted 1.96 for this study.

p = expected proportion in the study population based on previous studies or pilot studies, set at 80% for this study which was the national immunization coverage of the under-five-year-old children aged between 12-23 months [30].

d = absolute error or precision, set at 5% for this study. Premised on the above parameters, we computed our sample size for this study as follows:

N=(1.96^2^*0.80*0.20)/(0.05)^2^

N = 246

Notably, 5% (13) of the estimated sample size was considered in this study to increase precision, thus, 259 study participants were surveyed.

### Data collection and study variables

Data collection spanned from May to July 2019 using interviewer-administered structured questionnaires at the eleven public hospitals in Kiambu County by research assistants trained in data collection procedures as well as obtaining informed consent from the study participants (see extended data) [31,32]. We explained the purpose of the study to women with children seeking child welfare care at these facilities, and those who were eligible and agreed to participate in the study were invited to a separate room where informed consent was taken. Sample size estimates were allocated to each facility proportionate to the size of the number of women of reproductive with children aged under five years and seeking child welfare services at 9 o´clock during the data collection period (May to July 2019). Afterward, we recruited 259 study participants proportionate to size at each study hospital through systematic random sampling techniques following the selection of the first study participant by either a lottery technique or random number tables where applicable. Data quality was ensured by checking the completeness of the questionnaires filled in by the research assistants during the data collection process. We gathered data on the sociodemographic characteristics (maternal age, education, occupation, and gravidity) of the study participants. Further, we adopted and modified birth defects knowledge assessment questions developed by Bello AI *et al*. [33] to collect the additional data (see extended data) [34] (Table 1).

**Table 1:** study variables and their assessments

Variable (type)	Method of assessment
Maternal age (continuous)	Captured as a continuous variable in years.
Maternal education (nominal)	Captured as none, primary, secondary, college certificate, college diploma, and university degree.
Maternal occupation (nominal)	Captured as a nominal variable, categorized and labeled; employed, unemployed, and farming.
Gravidity	Captured as a continuous variable in weeks
Birth is acquired intrauterine (nominal)	Captured as a nominal variable, categorized and labeled; don’t know, no, and yes.
Cousin marriage may cause birth defects (nominal)	Captured as a nominal variable, categorized and labeled; don’t know, no, and yes.
Birth defects may be transmitted by infections (nominal)	Captured as a nominal variable, categorized and labeled; don’t know, no, and yes.
Birth defects are preventable (nominal)	Captured as a nominal variable, categorized and labeled; don’t know, no, and yes.

### Ethical considerations

Kenyatta National Hospital [KNH]-University of Nairobi [UoN] Ethics Review Committee provided ethical approval referenced KNH-ERC/A/44. National Commission for Science, Technology, and Innovation (NACOSTI) similarly permitted the study referenced P/19/75586/28325. We also got permission from Kiambu County Commissioner (ED.12(A)/1/VOL.11/107), and the Kiambu County Department of Health (KIAMBU/HRDU/AUTHO/2019/03/06/AgotGN). Similarly, informed consent was obtained from the study participants before the data collection procedures (see extended data [31].

### Statistical analysis

Data were entered in an Excel spreadsheet (see extended data [32] and exported to Stata software version 14.0 (Stata Corporation, Texas, USA) for cleaning, coding, and analyses. Descriptive analyses were conducted where categorical variables were presented as proportions and percentages in frequency tables, whilst continuous variables were presented in medians, means, standard deviations, and ranges. Each of the four-point birth defect knowledge assessment questions with responses of don´t know, no, and yes were computed at 100% to estimate the knowledge of the study respondents. Afterward, the estimates of each of the four knowledge questions were aggregated at 100% to estimate the overall average knowledge scores for the study participants.

## Results

**Sociodemographic characteristics of the study participants:** a total of 259 women of reproductive age were enrolled in this study [32] (Table 2). The median age of the study participants was 26 years with a mean of 27 (SD=5, Range=17-42). A majority (77%) achieved at least a secondary level of education, whilst the median gravidity was 2 with a mean of 2 (SD=1, Range=1-8) (Table 2).

**Table 2:** sociodemographic characteristics of the study participants (N=259)

Variables	Measurements	Frequency (n)	Percent (%)	Cumulative percent (%)
Maternal education	College certificate	10	3.86	3.86
	College diploma	60	23.17	27.03
	None	2	0.77	27.80
	Primary	57	22.01	49.81
	Secondary	127	47.88	97.68
	University	6	2.32	100.00
	**Total (N)**	**259**	**100**	
Maternal occupation	Employed	106	40.93	40.93
	Farmer	16	6.18	47.10
	Unemployed	137	52.90	100.00
	**Total (N)**	**259**	**100**	
Maternal age	Median	26		
	Mean	27		
	Standard deviation	5		
	Range	17-42		
	**Total (N)**	**259**		
Gravidity	Median	2		
	Mean	2		
	Standard deviation	1		
	Range	1-8		
	**Total (N)**	**259**	**100**	

**Birth defect knowledge assessment of the study participants:** approximately 22% of the study participants reportedly did not know that birth defects were acquired by infants intrauterine, whereas about 14% reported that birth defects were not acquired by infants in utero. Additionally, about 17% reported that they did not know that intrauterine infections could result in the occurrence of birth defects, whereas 39% and 45% reported that intrauterine infections could not cause birth defects and that intrauterine infections could cause birth defects, respectively (Table 3).

**Table 3:** birth defect knowledge assessment of the study participants (N=259)

Variables	Measurements	Frequency (n)	Percent (%)	Cumulative Percent (%)
A birth defect is acquired intrauterine	Don’t know	56	21.62	21.62
	No	36	13.90	35.52
	Yes	167	64.48	100.00
	**Total (N)**	**259**	**100**	
Intrauterine infection can cause birth defects	Don’t know	43	16.60	16.60
	No	100	38.61	55.21
	Yes	116	44.79	100.00
	**Total (N)**	**259**	**100**	
Some birth defects are preventable	Don’t know	10	3.89	3.89
	No	17	6.61	10.51
	Yes	230	89.49	100.00
	**Total (N)**	**257**	**100**	
Cousin marriage can cause birth defects	Don’t know	28	10.81	10.81
	No	57	22.01	32.82
	Yes	174	67.18	100.00
	**Total (N)**	**259**	**100.00**	

**Birth defect knowledge assessment scores:** nearly 13% of the study participants were not aware that birth defects could be acquired by the infant intrauterine, intrauterine infections could cause birth defects, some birth defects were preventable, and cousin marriages could also be responsible for the occurrence of birth defects. Additionally, close to 19% of the study participants reported that birth defects were not acquired before birth, births could not be caused by intrauterine infections, some birth defects could be prevented and cousin marriage could not cause birth defects. Nonetheless, a majority (67%) reported that births could be acquired intrauterine, infections intrauterine could cause birth defects, some birth defects could be prevented, and cousin marriages could also cause the occurrence of birth defects (Table 4).

**Table 4:** birth defect knowledge assessment scores (100%)

Variable	Don’t know (%)	No (%)	Yes (%)	Total Scores (%)
A birth defect is acquired intrauterine	21.62	13.90	64.48	100.00
Intrauterine infection can cause birth defects	16.60	36.61	44.79	100.00
Some birth defects are preventable	3.89	6.61	89.49	100.00
Cousin marriage can cause birth defects	10.81	22.01	67.18	100.00
**Total Scores**	**52.92**	**77.13**	**265.94**	**400.00**
**Average scores**	**13.23**	**19.28**	**66.49**	**100.00**

Average scores were computed by dividing individual scores by the number of (4) variables

## Discussion

Notably, almost (90%) of the study participants reported that birth defects could be prevented pointing to ease of comprehension of susceptibility, severity, and cues to action by women of reproductive. Additionally, the knowledge of the study participants was above average (67%). However, the study showed that approximately 11% of the study participants did not know that birth defects could be caused by consanguine marriages, whilst about 22% reported that consanguine marriage could not cause birth defects. This implied that about one-third (33%) of the study participants were not aware that cousin marriages could be responsible for the occurrence of birth defects. Further, this implied that for every 10 women of reproductive, three of them did not know that consanguine marriage could be responsible for birth defects occurrence. Even though consanguine marriages had been associated with birth defects of genetic origin that could not be physiologically modified, it could be of public health interest to create awareness among women of reproductive age that cousin marriages could result in birth defects arising from deoxyribonucleic acid variants physiologic interactions between the partners [1,3]. Specifically, offspring of cousin marriages could result in birth defects due to homozygosity of the deleterious recessive genes in heterozygous parents [34]. Raising awareness of consanguineous marriage would be significant in preventing and controlling the occurrence of birth defects particularly those of known genetic etiology.

Similarly, approximately one-third of the study participants did not know that birth defects were acquired by infants in utero, and did not know that the defects were acquired by infants intrauterine; an observation corroborated in the study conducted in Ghana by Bello and others [3,3]. Notably, the study noted that barely 45% of the study participants knew that intrauterine infections could cause birth defects. The occurrence of certain birth defects has been attributed to teratogenic gestational infections such as Rubella associated with deafness, blindness, cardiac defects, and nervous system defects [1,3,35]. Invariable effects of teratogenic infections on embryonic development could be reduced significantly if pregnant women would receive skilled antenatal care before eight weeks of gestation attributed to timely intake of folic acid, vitamin supplementation, treatment of teratogenic infections, and immunization against rubella [25,36-38].

Overall, the birth defect knowledge assessment showed that about one-third of the study, participants were not aware of the fact that birth defects were acquired intrauterine, attributed to some intrauterine infections, as well as consanguineous marriage, and that some of the birth defects could be prevented. It was important to note that the majority of the study participants had achieved at least a secondary level of education and thus would be able to comprehend health messages geared towards preventing and controlling the occurrence of birth defects. Additionally, the mean age, as well as the mean gravidity of the study participants pointed to the possible further gestations to achieve the fertility rate of 3.9 in Kenya further underscoring the importance of creating awareness of birth defects before subsequent pregnancies [30]. One of the limitations of this study was the number (four) of birth defect knowledge assessment questions. Nonetheless, the questions were precise and simple thus easily providing a snapshot of the level of birth defect knowledge of the study participants in the county.

## Conclusion

Even though women of reproductive had above-average knowledge of birth defects, they were similarly substantially deficient in birth defects knowledge in the county. Thus, we would like to recommend to public health policymakers and health care providers to formulate short health messages on birth defects tailored to women attending child welfare and antenatal clinics at all levels of health care including community health services in the county.

### What is known about this topic

- *Birth defects are malformations of prenatal origin detectable before birth, at birth, or later;*
- *Birth defects contribute to perinatal mortality globally*.

### What this study adds

- *It contributes to further understanding of the factors contributing to the occurrence of birth defects;*
- *Informs formulation of public health preventive strategies for the defects*.
